# Supplementary material for: Distinct causes underlie double-peaked trilobite morphological disparity in cephalic shape
Source: Commun Biol. 2024 Nov 12;7:1490. doi: 10.1038/s42003-024-07221-2 (PMC11557869; doi:10.1038/s42003-024-07221-2)
Supplement: Supplementary file 2 — Supplementary Information [file 42003_2024_7221_MOESM2_ESM.pdf]

Supplementary information for Drage & Pates, Distinct causes underlie double-peaked trilobite morphological disparity in cephalic shape

Supplementary Figure 1: K-means analysis displaying the natural clustering inherent in the trilobite cephalic outlines dataset.

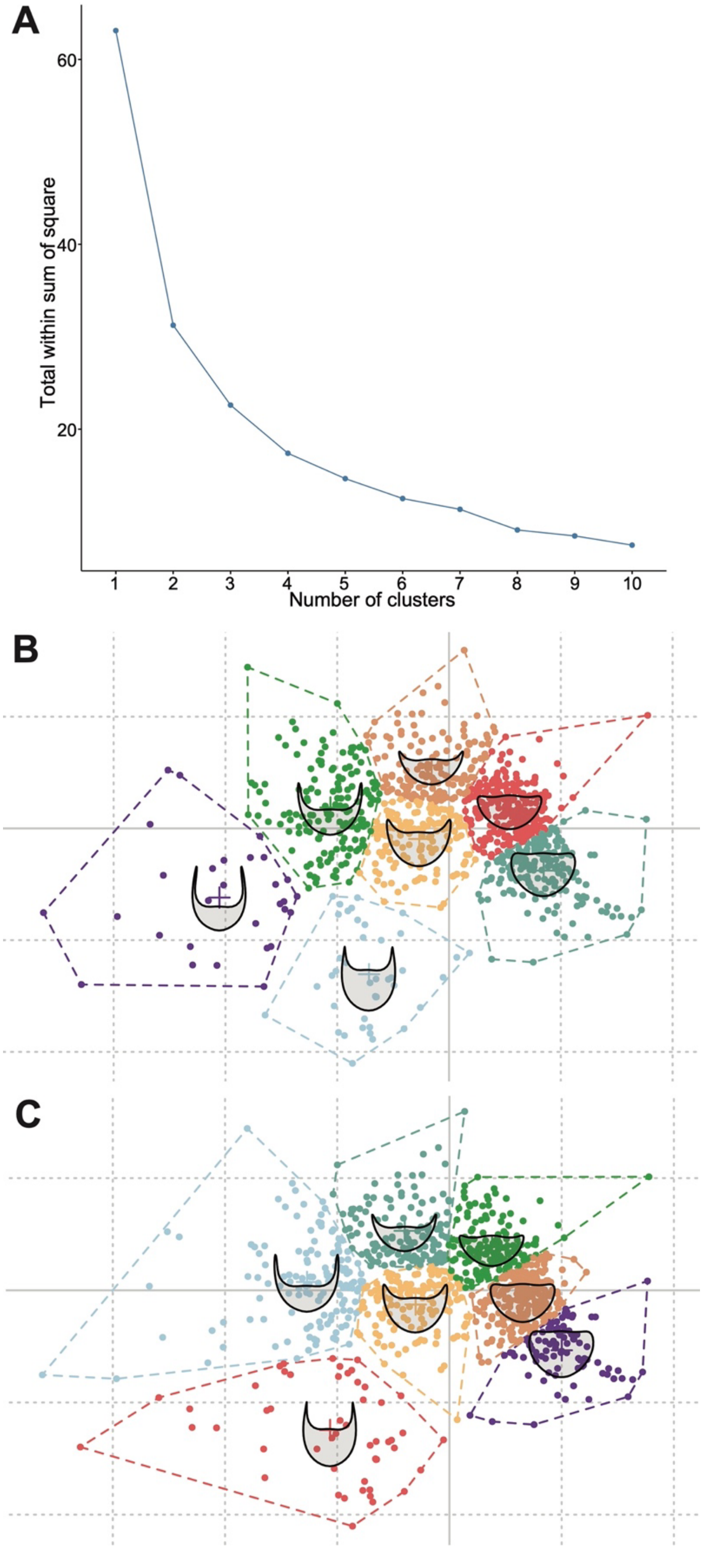

A, Elbow plot showing the most suitable number of clusters present in the dataset (7; the point at which there is a plateau followed by a clear down-trending slope on the y-axis). B and C, two, slightly differing, clustering hypotheses using k = 7 are present, which closely reflect all possible clustering hypotheses. N = 983 independent biological samples.

Supplementary Figure 2: Cephalic outline collection (data preparation phase) and analysis (data analysis phase) methodology.

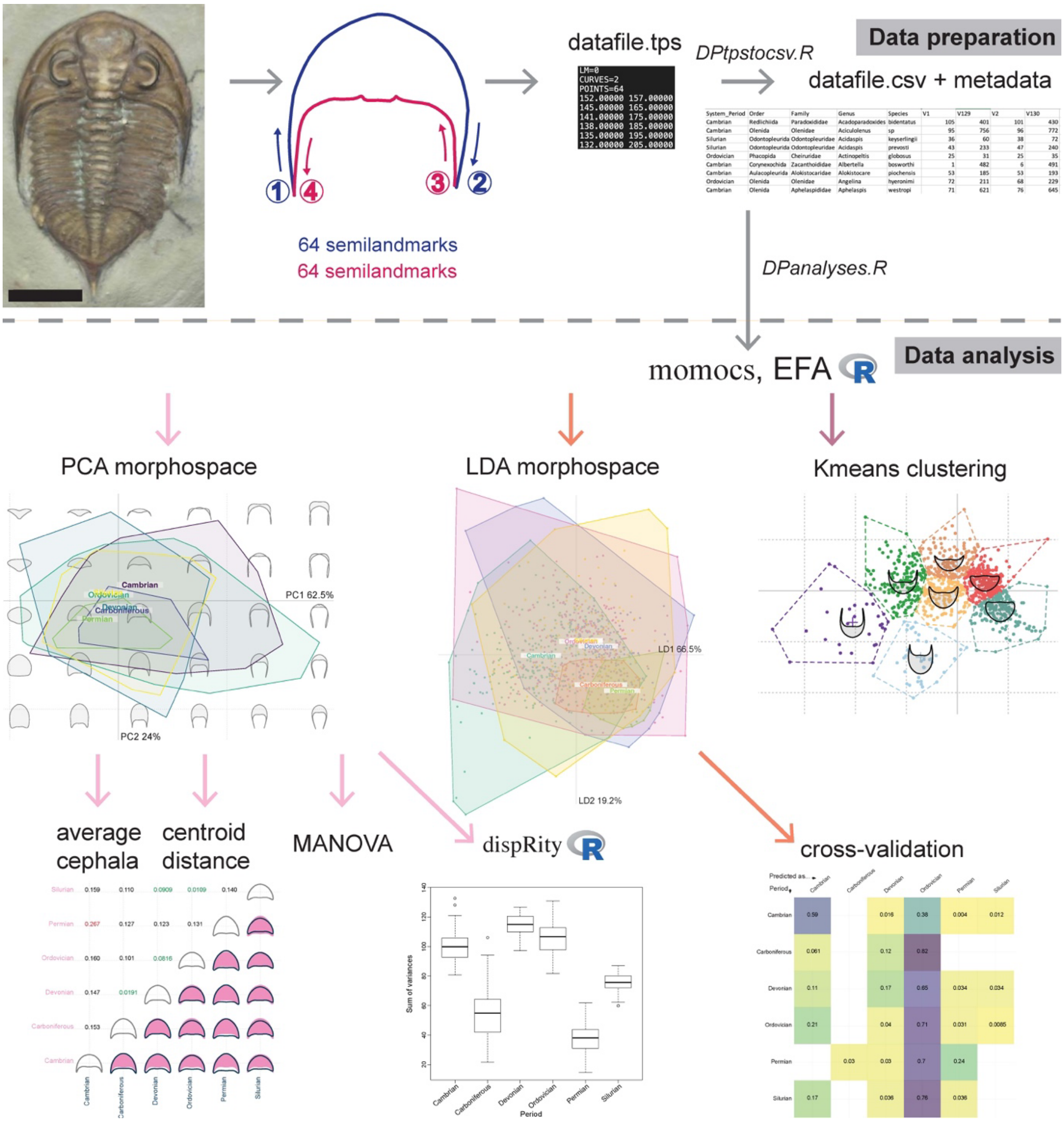

Numbers and arrows on the cephalon outline show the process of producing two semilandmark curves to capture the cephalon shape. Filenames given reflect the R code to run the various steps provided at https://osf.io/vz9a5/. EFA, elliptical Fourier transformation;

PCA, principal components analysis; LDA, linear discriminants analysis. Momocs R package by Bonhomme et al.[1], dispRity R package by Guillerme[2]. Trilobite image is a snapshot of a 3D model of *Dalmanites caudatus*, scale bar = 1 cm.

Tables

Supplementary Table 1: PCA morphospace areas occupied by the convex hulls of the taxonomic order groupings, and calculated disparity measures (sums of variances and sums of ranges; plotted in Fig. 5). N = 983 independent biological samples.

**Taxonomic order**	**Sample size (specimens)**	**Convex hull area (no unit)**	**Sums of variances (no unit)**	**Sums of ranges (no unit)**
Asaphida	115	0.31	0.065	1.534
Aulacopleurida	34	0.19	0.036	1.220
Corynexochida	68	0.29	0.045	1.425
Harpida	33	0.28	0.038	1.444
Lichida	21	0.16	0.048	1.089
Odontopleurida	23	0.18	0.043	1.029
Olenida	68	0.55	0.080	1.818
Phacopida	359	0.48	0.037	1.674
Proetida	100	0.25	0.034	1.211
Redlichiida	69	0.44	0.053	1.756
Trinucleida	38	0.51	0.133	2.023
Unassigned	55	0.31	0.043	1.436

Supplementary Table 2: PCA morphospace areas occupied by the convex hulls of the geological Period groupings, and calculated disparity measures (sums of variances and sums of ranges; plotted in Fig. 8). N = 983 independent biological samples.

**Geological Period**	**Sample size (specimens)**	**Convex hull area (no unit)**	**Sums of variances (no unit)**	**Sums of ranges (no unit)**
Cambrian	249	0.64	0.055	1.983
Ordovician	351	0.74	0.064	2.217
Silurian	169	0.40	0.047	1.556
Devonian	148	0.59	0.078	1.930
Carboniferous	33	0.14	0.029	0.995
Permian	33	0.096	0.021	0.831

1. Bonhomme, V., Picq, S., Gaucherel, C. & Claude, J. Momocs: outline analysis using R. *J. Stat. Softw.* **56**, 1–24. https://doi.org/10.18637/jss.v056.i13 (2014).

2. Guillerme, T. dispRity: A modular R package for measuring disparity. *Methods Ecol. Evol.* **9**, 1755–1763. https://doi.org/10.1111/2041-210X.13022 (2018).
