# Supplementary material for: Distinct causes underlie double-peaked trilobite morphological disparity in cephalic shape
Source: Commun Biol. 2024 Nov 12;7:1490. doi: 10.1038/s42003-024-07221-2 (PMC11557869; doi:10.1038/s42003-024-07221-2)
Supplement: Supplementary file 3 — Description of Additional Supplementary Materials [file 42003_2024_7221_MOESM3_ESM.pdf]

# Description of Additional Supplementary Files

**File name:** Supplementary Data I

**Description:** The raw source data used for all analyses and graphs in the paper (all outline landmarks for specimens, and the principle components coordinates used).

**File name:** Supplementary Data II

**Description:** The results of all statistical tests presented in the study (if not included in the manuscript), including all test values and p values, and multiple-test corrections.

**File name:** Supplementary Data III

**Description:** The search protocol and results used for gathering raw data from online sources.
