# Supplementary material for: Distinct causes underlie double-peaked trilobite morphological disparity in cephalic shape
Source: Commun Biol. 2024 Nov 12;7:1490. doi: 10.1038/s42003-024-07221-2 (PMC11557869; doi:10.1038/s42003-024-07221-2)
Supplement: Supplementary file 7 — Reporting Summary [file 42003_2024_7221_MOESM7_ESM.pdf]

nature portfolio

Corresponding author(s): Harriet B. Drage

Last updated by author(s): Nov 1, 2024

Reporting Summary

Nature Portfolio wishes to improve the reproducibility of the work that we publish. This form provides structure for consistency and transparency in reporting. For further information on Nature Portfolio policies, see our Editorial Policies and the Editorial Policy Checklist.

Statistics

For all statistical analyses, confirm that the following items are present in the figure legend, table legend, main text, or Methods section.

n/a	Confirmed	
☐	☒	The exact sample size (*n*) for each experimental group/condition, given as a discrete number and unit of measurement
☒	☐	A statement on whether measurements were taken from distinct samples or whether the same sample was measured repeatedly
☐	☒	The statistical test(s) used AND whether they are one- or two-sided<br>*Only common tests should be described solely by name; describe more complex techniques in the Methods section.*
☐	☒	A description of all covariates tested
☐	☒	A description of any assumptions or corrections, such as tests of normality and adjustment for multiple comparisons
☐	☒	A full description of the statistical parameters including central tendency (e.g. means) or other basic estimates (e.g. regression coefficient) AND variation (e.g. standard deviation) or associated estimates of uncertainty (e.g. confidence intervals)
☐	☒	For null hypothesis testing, the test statistic (e.g. *F*, *t*, *r*) with confidence intervals, effect sizes, degrees of freedom and *P* value noted<br>*Give P values as exact values whenever suitable.*
☒	☐	For Bayesian analysis, information on the choice of priors and Markov chain Monte Carlo settings
☒	☐	For hierarchical and complex designs, identification of the appropriate level for tests and full reporting of outcomes
☒	☐	Estimates of effect sizes (e.g. Cohen's *d*, Pearson's *r*), indicating how they were calculated

Our web collection on statistics for biologists contains articles on many of the points above.

Software and code

Policy information about availability of computer code

Data collection	tpsDIG2 open source software was used for data collection (semilandmark curves), in addition to freely available data previously published (Suarez & Esteve, 2021). This latter was resampled to give comparable data points using R, with the needed R code available open access at https://osf.io/vz9a5 (noted also in the manuscript methods). All semilandmark curves were obtained from photographs of fossil specimens (in which case, the specimen accession number is given in the supplementary data) or from the same from the descriptive literature (in which case, the relevant article is given in the supplementary data.
Data analysis	All analyses were performed using R version 2023.09.1+494, with code available open access at https://osf.io/vz9a5 (noted also in the manuscript methods and in the code availability statement).

For manuscripts utilizing custom algorithms or software that are central to the research but not yet described in published literature, software must be made available to editors and reviewers. We strongly encourage code deposition in a community repository (e.g. GitHub). See the Nature Portfolio guidelines for submitting code & software for further information.

Data

Policy information about availability of data

All manuscripts must include a data availability statement. This statement should provide the following information, where applicable:

- Accession codes, unique identifiers, or web links for publicly available datasets
- A description of any restrictions on data availability
- For clinical datasets or third party data, please ensure that the statement adheres to our policy

All data (the original data) used to produce the results reported in this manuscript are available in Supplementary Data I and open access at https://osf.io/vz9a5/. As above, this also contains the accession numbers and article links for the specimens. All R code needed to run the analyses are also available open access at https://osf.io/vz9a5/. Additionally, the specimen photos used to obtain the original semilandmarks are available online at the same link. A full data availability statement is present in the manuscript detailing all of these.

Research involving human participants, their data, or biological material

Policy information about studies with human participants or human data. See also policy information about sex, gender (identity/presentation), and sexual orientation and race, ethnicity and racism.

Reporting on sex and gender	Not applicable
Reporting on race, ethnicity, or other socially relevant groupings	Not applicable
Population characteristics	Not applicable
Recruitment	Not applicable
Ethics oversight	Not applicable

Note that full information on the approval of the study protocol must also be provided in the manuscript.

Field-specific reporting

Please select the one below that is the best fit for your research. If you are not sure, read the appropriate sections before making your selection.

☐ Life sciences ☐ Behavioural & social sciences ☒ Ecological, evolutionary & environmental sciences

For a reference copy of the document with all sections, see nature.com/documents/nr-reporting-summary-flat.pdf

Ecological, evolutionary & environmental sciences study design

All studies must disclose on these points even when the disclosure is negative.

Study description	We explore trilobite cephalic disparity using a dataset of 983 cephalon outlines, analysing the associations between cephalic morphometry and taxonomic assignment and geological Period. Elliptical Fourier transformation is carried out and visualised as a Principal Components Analysis, and, combined with Linear Discriminants Analysis, used to quantify morphospace occupation and disparity trends with geological time and taxonomy. Further analyses test explicitly the link between these factors and cephalic morphometry.
Research sample	The sample consists of a semilandmark outline for 597 trilobite cephalon fossil specimens, gathered from photographs of said specimens. These photographs were taken by the authors from accessioned museum specimens (Senckenberg Museum, Sedgwick Museum, Staatliches Museum fur Naturkunde Karlsruhe, Natural History Museum London - all data available in Supplementary Data I and online at https://osf.io/vz9a5/) or accessed through iDigBio (details of all searches available in supplementary material Supplementary Data II) or the published descriptive literature (see Supplementary Data I for sources). Additional cephalon outlines were sourced from the open access dataset published by Suarez and Esteve (2021), and resampled to ensure consistency of data with that collected by the authors.
Sampling strategy	Sampling was partially dictated by the fossil specimens available at the studied museum collections and available online (see above - due to the nature of fossil record sampling and collections availability). Sample sizes for geological periods and taxonomic groups were counted and deemed of sufficient size to include in analyses (all >20 specimens - see counts in tables 1 and 2). When calculating measures of disparity, bootstrapping was employed to address uneven sampling, and sample size was taken into account in all relevant discussion points and conclusions made in the manuscript.
Data collection	Cephalon outline semilandmark data were obtained from the photographs noted above using the free software tpsDIG2 - all semilandmark outlines were captured by Harriet B. Drage to ensure sampling consistency. Outlines consisted of two curves, one along the anterior cephalic margin and one the posterior, each consisting of 64 evenly spaced semilandmarks, giving 128

	semilandmarks total capturing each trilobite cephalon outline. The data published by Suarez and Esteve (2021) were resampled using R (code available) to give an equivalent number of semilandmarks for each outline. All metadata for trilobite specimens were recorded and checked by Harriet B. Drage and Stephen Pates using the original descriptive literature, geological Periods occupied given by Jell and Adrain (2002), and order assignment by Adrain (2011).
Timing and spatial scale	iDigBio data were collected during early 2021 (search terms and exact dates given in the supplementary.<br>Directly accessed museum data were gathered during late 2021 and 2022, entirely pertaining to when the authors were able to organise in-person visits to these collections (and partially mediated by covid restrictions). Descriptive literature photographs and the semilandmark data sampling were carried out across 2022 by Harriet B. Drage, but this data-gathering is unrelated to timing.<br>Given the nature of the palaeontological data, sample cohorts divided based on sampling timing are inapplicable.
Data exclusions	Data pertaining to agnostids were excluded from the dataset, due to the ongoing debate over their taxonomic position (within or outside Trilobita).
Reproducibility	All experimental findings were confirmed by rerunning all analyses multiple times using R, with no differences obtained between these tests. This excepts those analyses that provide stochastic explorations of the data (e.g., kmeans clustering on the dataset) - in this case, the analysis was rerun for a minimum of 20 times subsequently, and the two hypothetical results that consistently appeared time and again were taken and both hypotheses presented in the study.
Randomization	Allocation was not randomised, as the groupings were the factors explicitly to be tested in the study.
Blinding	Blinding is not relevant to this study - there is no human data, nor data that could be biased by human perspectives.

Did the study involve field work? ☐ Yes ☒ No

Reporting for specific materials, systems and methods

We require information from authors about some types of materials, experimental systems and methods used in many studies. Here, indicate whether each material, system or method listed is relevant to your study. If you are not sure if a list item applies to your research, read the appropriate section before selecting a response.

Materials & experimental systems

n/a	Involved in the study	
☒	☐	Antibodies
☒	☐	Eukaryotic cell lines
☐	☒	Palaeontology and archaeology
☒	☐	Animals and other organisms
☒	☐	Clinical data
☒	☐	Dual use research of concern
☒	☐	Plants

Methods

n/a	Involved in the study	
☒	☐	ChIP-seq
☒	☐	Flow cytometry
☒	☐	MRI-based neuroimaging

Palaeontology and Archaeology

Specimen provenance	No permits were required because all fossil specimens analysed for the study are publicly accessible in museum collections - the specimen accession details are given in the supplementary information and online (see above).
Specimen deposition	Specimens photographed by the authors are publicly deposited at the Senckenberg Museum, Sedgwick Museum, Staatliches Museum fur Naturkunde Karlsruhe, and Natural History Museum London - see specimen information in the supplementary information. Images obtained from the literature for data collection are traceable by their references, given in the supplementary information. Images obtained from iDigBio are available open online, with search terms used to access them given in the supplementary information.
Dating methods	Not applicable.

☐ Tick this box to confirm that the raw and calibrated dates are available in the paper or in Supplementary Information.

Ethics oversight	No ethical approval or guidance was required - all data obtained are from publicly accessible and accessioned museum of specimens of palaeontological material.

Note that full information on the approval of the study protocol must also be provided in the manuscript.

Plants

Seed stocks	N/A
Novel plant genotypes	N/A
Authentication	N/A
